# Acute Thiopurine Overdose: Analysis of Reports to a National Poison Centre 1995–2013

**DOI:** 10.1371/journal.pone.0086390

**Published:** 2014-01-29

**Authors:** Claudia Gregoriano, Alessandro Ceschi, Christine Rauber-Lüthy, Hugo Kupferschmidt, Nicholas R. Banner, Stephan Krähenbühl, Anne B. Taegtmeyer

**Affiliations:** 1 Department of Clinical Pharmacology and Toxicology, University and University Hospital Basel, Basel, Switzerland; 2 University Clinic of Internal Medicine, Kantonspital Baselland Liestal, Liestal, Switzerland; 3 Swiss Toxicological Information Centre, Associated Institute of the University of Zurich, Zurich, Switzerland; 4 Department of Clinical Pharmacology and Toxicology, University Hospital Zurich, Zurich, Switzerland; 5 The Royal Brompton and Harefield NHS Foundation Trust, Harefield Hospital, Harefield, Middlesex, United Kingdom; 6 National Heart and Lung Institute and Institute of Cardiovascular Medicine and Research, Imperial College, London, United Kingdom; National Institutes of Health, United States of America

## Abstract

Literature regarding acute human toxicity of thiopurines is limited to a handful of case reports. Our objectives were to describe all cases of overdose with thiopurines reported to the Swiss Toxicological Information Centre between 1995–2013. A retrospective analysis was performed to determine circumstances, magnitude, management and outcome of overdose with these substances. A total of 40 cases (14 paediatric) were reported (azathioprine, n = 35; 6-mercaptopurine, n = 5). Of these, 25 were with suicidal intent, 12 were accidental and 3 were iatrogenic errors. The magnitude of overdose ranged from 1.5 to 43 (median 8) times the usual dose in adults. Twelve cases (30%) had attributable symptoms. The majority of these were minor and included gastrointestinal complaints and liver function test and blood count abnormalities. Symptoms were experienced by patients who took at least 1.5-times their usual daily thiopurine dose. Overdoses over two or more consecutive days, even if of modest size, were less well tolerated. One case of azathioprine and allopurinol co-ingestion over consecutive days led to agranulocytosis. Decontamination measures were undertaken in 11 cases (10 activated charcoal, 1 gastric lavage) and these developed fewer symptoms than untreated patients. This study shows that acute overdoses with thiopurines have a favourable outcome in the majority of cases and provides preliminary evidence that gastrointestinal decontamination with activated charcoal may reduce symptom development after overdose of these substances if patients present to medical services soon after ingestion.

## Introduction

Thiopurines are immunosuppressant drugs used in the treatment of autoimmune conditions and acute leukaemias as well as in the prevention of acute rejection after solid organ transplantation. Azathioprine (AZA), 6-mercaptopurine (6-MP) and thioguanine are the thiopurines currently in use and have been licensed in Switzerland for 48, 58 and 40 years, respectively. AZA is a precursor of 6-MP which in turn is a precursor of thioguanine. Being purine antimetabolites, thiopurines prevent normal cell development and division of rapidly expanding cell lines such as haematological cell lines which leads to their desired immunosuppressive effects as well as to toxicity. Patients taking these agents or their household contacts may be intentionally or accidentally exposed to overdose. Despite several decades of experience in using thiopurines and a good knowledge about long-term toxicity, the published information regarding acute toxicity in overdoses only consists of a small number of case reports from different centres [1]–[6].

Gastrointestinal symptoms such as nausea, vomiting, abdominal pain and diarrhoea appear to be the commonest early initial features of acute toxicity, followed by subsequent reversible liver function test and haematological abnormalities, however the collective experience is limited. Furthermore little is known about the circumstances and optimal management of thiopurine overdose – a condition for which no specific antidotes are available. The purpose of this study was to investigate the circumstances, management and outcomes of overdoses with thiopurines using data reported to a single national poison centre during an 18-year period.

## Materials and Methods

### Study design

A specific ethics approval was not required for this observational study due to the nature of the study design according to the regulations of the cantonal ethics committee Zurich, Switzerland (www.kek.zh.ch/) which also state that anonymised data generated during patient care can be used retrospectively for research purposes without obtaining written consent. We performed a retrospective review of all acute overdoses involving thiopurines in adults and children (<16 years) either alone or in combination with other drugs that had been reported to the Swiss Toxicological Information Centre (STIC) between April 1995 and August 2013 (for further details, please see the online Supporting Information Methods S1). Cases are assigned an internal identification number and neither the patients nor the reporting professionals could be identified by the investigators.

### Circumstances and symptoms of overdose

The circumstances of overdose were categorised as ‘suicidal’ for cases of intentional overdose, ‘domestic’ for cases of accidental overdose in the home and ‘iatrogenic’ for those due to a prescribing or administration error in hospital.

The severity of symptoms were graded in accordance with the Poisoning Severity Score (PSS) as ‘minor’, for mild, transient and spontaneously resolving symptoms/signs; ‘moderate’, if at least one pronounced or prolonged symptom/sign was recorded; ‘severe’, if at least one severe or life-threatening symptom/sign was observed, or ‘fatal’, if the overdose was the recorded cause of death [7].

Cases were assessed for association between symptoms and the immunosuppressant overdose by an expert panel including a senior clinical pharmacologist and a senior clinical toxicologist, both with additional qualifications in general internal medicine, using the World Health Organisation Uppsala Monitoring Centre (WHO-UMC) standardised case causality assessment criteria originally developed for the assessment of adverse drug reactions [8]. Co-morbidities, co-ingestion of other medication (in patients with multiple drug overdose or taking other drugs in the therapeutic dose range) and the magnitude of overdose were taken into consideration. Associations were classified as ‘certain’, ‘likely’, ‘possible’ and ‘unlikely’ (Table S1).

### Statistical analyses

Descriptive statistics were used to analyse grouped data. Overdoses in mg/kg were compared with usual therapeutic doses by determining the multiple of the subject's usual therapeutic dose (dose ingested/usual dose). In subjects who did not normally receive a thiopurine or in cases where the usual maintenance dose was not known, the magnitude of overdose above the maximum licensed dose was determined. In Switzerland the maximum licensed doses for oral AZA is 5 mg/kg/day (adults and children), and for oral 6-MP 2.5 mg/kg/day (adults and children) [9]–[10]. Missing data regarding patient weight was computed as detailed in the online Supporting Information (Methods S1).

## Results

### Cases

There were a total of 152762 reports to the STIC of confirmed or suspected overdose with any substance by healthcare professionals during the study period. Of these, 35 were with AZA and five with 6-MP. There were no overdoses with thioguanine. Thirty-five cases were reported by hospital doctors and five by other doctors. Demographic characteristics, circumstances of overdose, the magnitude of overdose and the availability of follow-up data are given in Table 1. Adults and children are presented separately. Table S2 shows the details of each individual case. Nine of the total 12 accidental domestic overdoses were in children under the age of four living with parents or siblings undergoing treatment with thiopurines.

**Table 1:** Demographic characteristics, circumstances, magnitude of overdose and follow-up data of the cases.

	Adults	Children
**Number** (%)	26 (65%)	14 (35%)
**Azathioprine**	26 (100%)	9 (64%)
**6-mercaptopurine**	0	5 (36%)
**Male** (%)	12 (46%)	9 (64%)
**Body mass range (kg)** (n for which data available)	50–80 (13)	11–48 (14)
**Access to immunosuppression:**		
Condition requiring immunosuppression (%)	18 (69%)	3 (21%)
No underlying condition requiring immunosuppression (%)	1 (4%)	10 (72%)
Unknown	7 (27%)	1 (7%)
**Circumstances of overdose**		
Suicidal intent	22 (85%)	3 (21%)
Domestic	1 (4%)	11 (79%)
Iatrogenic	3 (11%)	0
**Overdose with more than one substance**	20 (77%)	3 (21%)
**Median overdose as a multiple of usual ingested dose or maximum licensed dose** (range), (number for which data available)	8 (1.5–49) (25)	3 (0.22–13) (14)
**Median time (h) until contact with poisons information centre** (range) (number for which data available)	4 (<1–225) (23)	<1 (<1–8) (13)
**Number of cases for whom follow-up available**	14	7
**Median follow-up time** (range) (days)	4 (1–24)	5 (1–30)

Data was available for all cases unless otherwise specified.

Table S3 shows the magnitude of overdose in relation to the patient's usual maintenance dose or - for treatment-naïve individuals - in relation to the maximum licensed dose for each case. Four of the exposures (all with AZA) were retrospectively found to be less than the maximum licensed dose and were all cases of accidental ingestion by children. These cases were considered ‘accidental exposures within the therapeutic range’ rather than ‘overdose’ and were not included in the analysis of symptom development in order to accurately reflect the effect of true overdose.

There were three instances in which patients were exposed to ‘repeated overdoses’. Patient 29 had been co-treated with allopurinol for a number of days, patient 34 took an overdose of the same size on the two consecutive evenings prior to seeking medical attention and patient 35 unintentionally took double his prescribed dose for three days prior to presentation.

### Outcomes

The outcomes of overdose are given in detail in Table S3. None of the 6-MP overdoses caused symptoms. AZA was judged to have caused or contributed to symptoms or abnormal clinical findings in 12 of the 31 true overdose cases. The four children not usually taking AZA who were exposed to it in the therapeutic range did not develop symptoms. Symptoms attributable to AZA overdose were nausea and vomiting (n = 5), abdominal pain (n = 3), palpitations or sinus tachycardia (n = 2), headache (n = 2) and dyspnoea (n = 1). Laboratory findings attributable to thiopurine ingestion were increased liver enzymes (n = 3) and changes in blood count (n = 2). The symptom severities were classified as follows: 14 minor, three moderate and one severe. The severe case (patient 29) had undergone co-therapy with AZA and allopurinol prior to presenting with sepsis secondary to agranulocytosis. The duration of the co-therapy and the doses in this case are unknown. The three cases in which AZA was taken in overdose over two or more days (in one case in the context of the above-mentioned drug-drug interaction) all developed symptoms or signs attributable to AZA. No patients who ingested 1.5 times or less of their usual or the maximum licensed AZA dose developed symptoms. Patients who developed symptoms after overdose on a single occasion had been exposed to a mean of 14.1±11 times their usual or maximum licensed dose compared to asymptomatic patients who were exposed to 7.9±8.8 times their usual or maximum licensed dose.

### Management

In total, 4 patients were discharged immediately after initial evaluation in the emergency room, 5 were admitted for brief observation and discharged on the day of presentation, and 10 were admitted to acute medical wards. Hospitalisation status was unknown in 21 patients. Of those hospitalised, median length of stay was two days (range 1–11). Care was transferred from acute medical to psychiatric in-patient services in 6 cases. Among the subgroup of monointoxications for whom data were available, four were discharged on the same day, one patient after three days, one after more than eight days (exact figure unknown) and one after 11 days; all were asymptomatic.

The recommendation for use and subsequent use of decontamination measures is shown in Table S3. In 11 (28%) cases measures such as administration of activated charcoal (n = 9), induction of vomiting by ipecac syrup and activated charcoal administration (n = 1), or gastric lavage (n = 1) were performed. No decontamination measures were undertaken in 14 (35%) patients and in 15 (38%) it is not known if any action was performed. Haemodialysis was only proposed in patient 29 who had received AZA and allopurinol co-therapy. Gastrointestinal decontamination was performed more often in paediatric patients: 8 underwent it, 1 did not, and data were unavailable for five. Among the adults, these figures were 3, 13 and 10, respectively. Decontamination was performed in 1 case of accidental exposure within the therapeutic range and recommended in another case. Blood counts were performed in 11 (28%) cases (median four days after overdose, range 0.25–10 days) and liver function tests in 10 (25%) (median four days after overdose, range 0.25–10 days).

### Effect of decontamination

Fewer patients who received activated charcoal or underwent gastric lavage after overdose developed symptoms compared to patients in whom gastrointestinal decontamination was not performed. Nine of ten (90%) patients who underwent gastrointestinal decontamination after overdose remained asymptomatic compared to seven of the 14 (50%) who did not undergo gastrointestinal decontamination. No patients with 6-MP overdose developed symptoms, however follow-up was only one hour in the cases who did not receive decontamination. The mean multiple above patients' usual dose or maximum licensed dose of thiopurines were similar in the treated and untreated patients (10.68±12.63 and 10.42±10.5 respectively) while the mean times between overdose and presentation to medical services were approximately 2.8 hours (median 1.5, range <1–12 h) and 23 hours (median 2.25, range <1–96 h) respectively. No adverse effects resulting from treatment with activated charcoal or gastric lavage were reported.

## Discussion

This retrospective analysis of thiopurine overdoses reported to a single national poison centre between 1995 and 2013 adds a further 40 cases to the eight case reports currently published in the medical literature [1]–[6]. A large dataset such as the one presented here allows patterns, outcomes and management strategies of thiopurine overdoses to be more comprehensively assessed.

### Circumstances of overdose

The majority of overdoses in adult patients were with suicidal intent (usually in conjunction with other medication), whereas accidental overdoses were the most common among children. The current literature includes four adult cases, none of which were with suicidal intent and four paediatric cases [1]–[6]. Three of the adult cases were iatrogenic where 6-MP had been dispensed instead of propylthiouracil [3], [6] and the remaining case was accidental due to failure to understand the prescriber's instructions [1]. Among published cases in children, two were with suicidal intent [2], [5] and two were accidental ingestions by siblings of children receiving 6-MP [4]. While the majority of adults who took thiopurine overdoses with suicidal intent in the current series were themselves patients suffering from a chronic disease requiring immunosuppression, there were at least two cases where suicide was attempted by taking an overdose of a household contact's medication. Similarly, nine of the 11 accidental ingestions in children also involved a household contact's medication as in the two cases reported by Chow and colleagues [4]. Such cases further emphasise the need to repeatedly instruct patients to keep medicines out of reach of unintended recipients.

In contrast to the previously published literature about thiopurine overdoses and our findings for calcineurin inhibitor overdoses, where iatrogenic errors accounted for 38% and 46% respectively of the reported cases [1]–[6], [11], iatrogenic dosing errors only accounted for 8% of the current series. This may be due to the fact that the trade names for propylthiouracil and 6-MP in Switzerland are Propycil® and Puri-Nethol® respectively, which are more markedly different than in the USA where the mix-up errors occurred (Propylthiouracil® and Purinethol® respectively) [3], [6]. Furthermore, intravenous and liquid oral formulations, which accounted for almost three quarters of the CNI iatrogenic overdoses, are much less commonly used for the administration of thiopurines.

### Symptoms

Symptoms occurring during the first few hours after thiopurine overdose were nausea, vomiting, abdominal pain, palpitations or tachycardia, and headache. Other than tachycardia and palpitations, these symptoms have been noted in other cases of overdose [1], [2] or are reported in the product information for AZA and 6-MP [9], [10]. AZA has been associated with bradycardia [12] but not with tachycardia before. In the two cases of tachycardia we report, despite there being a good temporal correlation between AZA exposure and symptom development and no other concurrent drugs taken in overdose, the tachycardia may have additionally been caused by the circumstances of presentation (hospitalisation after a suicide attempt). We therefore judged the association to be ‘possible’ in accordance with the WHO causality criteria.

The weight-adjusted exposures in the three patients who developed blood count and/or liver function test changes and for whom complete data were available were 180 mg/kg (patient 19), 33 mg/kg (patient 28) and 18.6 mg/kg (patient 1). All of these overdoses were well below the median lethal dose (LD50) of AZA in mice (2500 mg/kg) and rats (400 mg/kg) [13] which might explain the paucity of severe symptoms seen in ours and the reported cases. Due to packaging size, patients are unlikely to have access to a dose which would cause severe toxicity if taken on a single occasion. This is in contrast to other drugs commonly taken in overdose such as acetaminophen, benzodiazepines and opiates.

Our findings and those previously published in the literature suggest that single overdoses of thiopurines are generally well tolerated [1], [2], [4], [5]. Sustained overdoses over two or more days, even if modest in size, however, are less well tolerated, as we found in three cases and as is also reported in the literature [3], [6]. This discrepancy in findings between acute and sustained overdoses is likely due to the relatively short half-lives of AZA and 6-MP (4.5 and 0.9 hours respectively), which means that nearly all of the parent compound will have been eliminated approximately 23 and 5 hours respectively after overdose on a single occasion. The development of severe symptoms after co-therapy with AZA and allopurinol can be explained by the inhibition of xanthine oxidase by allopurinol. This leads to reduced conversion of the active 6-MP into the inactive 6-thiouric acid metabolite and consequently to myelosuppression unless the thiopurine dose is reduced to one quarter of the usual daily recommended dose [9].

### Management

Patients who ingested less than 1.5 times their usual or the maximum recommended thiopurine dose did not develop symptoms and medical treatment or hospitalisation of such cases (other than for psychiatric reasons) does not appear to be indicated.

The Swiss product information for AZA and 6-MP does not recommend the use of activated charcoal after overdose [9], [10]. Other sources, however, recommend that activated charcoal be used in overdose of these drugs [12]. When administered correctly, activated charcoal is more effective, safer and more easily tolerated than gastric lavage, which it has largely replaced in recent years [14]. Due to the rarity of thiopurine overdose, it is not possible to perform randomised controlled trials to determine the efficacy of activated charcoal, and examination of case data such as ours is therefore important. Interestingly, we found that activated charcoal use in thiopurine overdose was more often associated with asymptomatic courses than seen in untreated cases, suggesting a benefit. On the basis of their chemical properties, a good adsorption of thiopurines onto activated charcoal is to be expected. AZA and 6-MP have molecular weights of 277 and 152 Dalton respectively, meaning that they are readily adsorbed into the 10–20 Å-sized charcoal pores [15]. The bioavailability of AZA is high (88%), whereas that of 6-MP is low (16%, reduced to 11% if ingested with food) and both reach their peak plasma concentrations within 2 hours of ingestion [9], [10]. It is therefore reasonable to expect that activated charcoal is beneficial when administered within 2 hours after overdose. However, activated charcoal should be restricted to cases of definite overdose as its administration is not without risk [16]. The fact that activated charcoal was administered to children more often than to adults possibly reflects their earlier presentation to emergency services and the difficulty medical staff have in obtaining an accurate account of the amount of drug ingested.

Prolonged hospitalisation for observation after thiopurine overdose does not routinely seem warranted on the basis of the data we present here. The maximum effect on the bone marrow is usually seen after 9 to 14 days [9], so follow-up blood counts are only meaningful when performed in this time period. However, no cases of subsequent bone marrow or liver failure were reported in this current large case series. This calls into question the need for follow-up blood tests after acute overdose. As an alternative, patients or caregivers could be instructed to represent themselves to medical services if they begin to feel unwell or develop fever.

In patients normally taking AZA or 6-MP who have been exposed to overdose, the decision about when these should be restarted should take the current clinical situation (in particular blood counts and liver function tests), the underlying disease activity and the relatively short half-lives of the compounds into consideration. The case of a single 50-fold AZA overdose reported by Carney showed a second leukopenic episode when therapy was reintroduced one week after overdose, so careful follow-up after reintroduction of thiopurines after very large overdoses seems prudent.

### Limitations

It is likely that our data did not capture all cases of overdose which occurred in the referral population. Our data are also incomplete, which is the nature of retrospective studies using poison centre data [17]. Unfortunately, a number of cases were lost to follow-up after the initial contact, despite repeated efforts by the STIC to contact the treating physicians in the days after presentation. In some cases follow-up took place sooner than 10 days after overdose which is when bone marrow suppression after ingestion of a thiopurine might be expected. A further limitation is the lack of data regarding thiopurine blood or plasma concentrations which would have allowed conclusions regarding pharmacokinetic-pharmacodynamic relationships and the effect of decontamination on drug absorption to be drawn. However, such data are not available as drug and metabolite concentrations for thiopurines are not routinely measured.

In conclusion, acute thiopurine overdose occurred commonly in the context of suicide attempt among adults and accidental poisoning in the home among children. The acute overdoses were well tolerated. In contrast, overdose over two or more consecutive days, even if of modest size, was associated with abdominal symptoms and liver function test and blood count abnormalities. Gastrointestinal decontamination with activated charcoal appeared to reduce symptom development after acute overdose and so should always be considered in cases presenting to emergency services within 2 hours. Thiopurine overdose patterns, outcomes and management require continued study and clinical toxicologists, clinical pharmacologists and treating physicians should be encouraged to actively seek follow-up data on the cases with which they are involved.

## Supporting Information

Methods S1 (DOC)

Table S1 **World Health Organisation Uppsala Monitoring Centre (WHO-UMC) causality categories** [**8**] **.**
(DOCX)

Table S2 **Patient demographics, circumstances and size of overdose.**
(DOCX)

Table S3 **Overdosage expressed as a multiple of the patient's usual dose (or factor above maximum licensed dose), decontamination measures and clinical findings.**
(DOCX)
