# Supplementary material for: Acute Thiopurine Overdose: Analysis of Reports to a National Poison Centre 1995–2013
Source: PLoS One. 2014 Jan 29;9(1):e86390. doi: 10.1371/journal.pone.0086390 (PMC3906026; doi:10.1371/journal.pone.0086390)
Supplement: Methods S1 — (DOC) [file pone.0086390.s001.doc]

**Supplemental Methods S1**

**Acute thiopurine overdose: analysis of reports to a national poison centre 1995 - 2013**

Claudia Gregoriano^*^, Alessandro Ceschi^*^, Christine Rauber-Lüthy, Hugo Kupferschmidt, Nicholas R. Banner, Stephan Krähenbühl, Anne B. Taegtmeyer

^*^ joint authors

Corresponding author: A. B. Taegtmeyer Department of Clinical Pharmacology and Toxicology, University and University Hospital Basel, Basel, Switzerland [anne.taegtmeyer@usb.ch](mailto:anne.taegtmeyer@usb.ch)

**Materials and Methods**

*Swiss Toxicological Information Centre operational procedures and data collection methods*

The Swiss Toxicological Information Centre (STIC) provides 24-hour 7-days-a-week nationwide free medical advice to healthcare professionals and the general public (referral population approximately 7.9 million) for the management of cases of human poisoning by any substance. Demographic and detailed clinical information on exposure cases such as age, weight, and sex of the patient, circumstances of the poisoning, ingested doses of all substances involved, symptoms and advice provided are recorded in a standardized manner by clinical toxicologists who are blinded to any study hypotheses. Data are anonymized and prospectively entered into an in-house database. Follow-up data including drug concentrations, therapeutic interventions and any decontamination measures which were performed and clinical course and complications are collected using standardized report forms sent to the treating physicians in the days after the initial contact.

*Calculation of missing body weight data*

Missing data regarding patient weight was computed using the mean weight for adult men and women. For children the mean weight of patients nearest in age was used. As these three categories were individually relatively small, a wider population of all calcineurin inhibitor, azathioprine, mycophenolate, 6-mercaptopurine, and sirolimus overdose cases reported to STIC in the same time frame (n=76) were examined for the purposes of determining mean population weights. The average adult male weight was 69.4 kg; for adult females this figure was 55.8 kg.
