# Supplementary material for: Acute Thiopurine Overdose: Analysis of Reports to a National Poison Centre 1995–2013
Source: PLoS One. 2014 Jan 29;9(1):e86390. doi: 10.1371/journal.pone.0086390 (PMC3906026; doi:10.1371/journal.pone.0086390)
Supplement: Table S1 — World Health Organisation Uppsala Monitoring Centre (WHO-UMC) causality categories [8] . (DOCX) [file pone.0086390.s002.docx]

**Table S1** World Health Organisation Uppsala Monitoring Centre (WHO-UMC) causality categories (Reference S1)

| **Causality term** | **Assessment criteria*** |
| --- | --- |
| **Certain** | • Event or laboratory test abnormality, with plausible time relationship to drug intake |
|  | • Cannot be explained by disease or other drugs |
|  | • Response to withdrawal plausible (pharmacologically, pathologically) |
|  | • Event definitive pharmacologically or phenomenologically (i.e. an objective and specific medical disorder or a recognised pharmacological phenomenon) |
|  | • Rechallenge satisfactory, if necessary |
| **Probable/Likely** | • Event or laboratory test abnormality, with reasonable time relationship to drug intake |
|  | • Unlikely to be attributed to disease or other drugs |
|  | • Response to withdrawal clinically reasonable |
|  | • Rechallenge not required |
| **Possible** | • Event or laboratory test abnormality, with reasonable time relationship to drug intake |
|  | • Could also be explained by disease or other drugs |
|  | • Information on drug withdrawal may be lacking or unclear |
| **Unlikely** | • Event or laboratory test abnormality, with a time to drug intake that makes a relationship improbable (but not impossible) |
|  | • Disease or other drugs provide plausible explanations |
| **Conditional/** | • Event or laboratory test abnormality |
| **Unclassified** | • More data for proper assessment needed, or |
|  | • Additional data under examination |
| **Unassessable/** | • Report suggesting an adverse reaction |
| **Unclassifiable** | • Cannot be judged because information is insufficient or contradictory |
|  | • Data cannot be supplemented or verified |

*All points should be reasonably complied with
