# Supplementary material for: Acute Thiopurine Overdose: Analysis of Reports to a National Poison Centre 1995–2013
Source: PLoS One. 2014 Jan 29;9(1):e86390. doi: 10.1371/journal.pone.0086390 (PMC3906026; doi:10.1371/journal.pone.0086390)
Supplement: Table S2 — Patient demographics, circumstances and size of overdose. (DOCX) [file pone.0086390.s003.docx]

**Table S2** Patient demographics, circumstances and size of overdose

| **Patient** | **Age** | **Sex** | **Weight (kg)** | **Underlying condition** | **Circumstance of overdose** | **Mono-Intoxication** | **Drug** | **Dose (mg)** | **Dose (mg/kg)^a^** | **Subject's usual dose (mg/d)** |
| --- | --- | --- | --- | --- | --- | --- | --- | --- | --- | --- |
| **Oral Azathioprine overdose** | | | | | | | | | | |
| 1^b^ | 13 | m | 35 | Chronic polyarthritis | Suicidal | Y | AZA tablets | 650 | 18.6 | 50 |
| 2 | 30 | m | 80 | Inflammatory bowel disease | Suicidal | N | AZA tablets | 500 | 6.3 | 50 |
| 3 | adult | f | uk | Unknown condition requiring immunosuppression | Iatrogenic | Y | AZA tablets | 150 | 2.7 | 100 |
| 4 | 39 | f | 50 | Heart transplant | Suicidal | N | AZA tablets | 800 | 16.0 | uk |
| 5 | adult | m | uk | Heart transplant | Suicidal | N | AZA tablets | 525 | 7.6 | uk |
| 6 | adult | f | uk | Unknown diagnosis | Suicidal | N | AZA tablets | 2500 | 44.8 | uk |
| 7 | 2.5 | m | 14 | None | Domestic | Y | AZA tablets | 50 | 3.6 | 0 |
| 8 | 25 | m | 80 | Unknown diagnosis | Suicidal | Y | AZA tablets | 625 | 7.8 | uk |
| 9 | 14 | f | 48 | Unknown diagnosis | Suicidal | N | AZA tablets | 1800 | 37.5 | uk |
| 10 | 30 | m | uk | Unknown diagnosis | Suicidal | N | AZA tablets | 2500 | 36.0 | uk |
| 11 | 25 | f | uk | Unknown diagnosis | Suicidal | N | AZA tablets | 2500 | 44.8 | uk |
| 12 | 43 | f | uk | Behçet`s disease, depression, fibromyalgia | Suicidal | N | AZA tablets | 1200 | 21.5 | 150 |
| 13 | 23 | f | 63 | Inflammatory bowel disease | Suicidal | N | AZA tablets | 750 | 11.9 | uk |
| 14 | 45 | m | 60 | Inflammatory bowel disease, depression, type 2 diabetes mellitus | Suicidal | N | AZA tablets | 900 | 15.0 | 100 |
| 15 | adult | f | uk | Kidney - pancreas transplant | Suicidal | N | AZA tablets | 900 | 16.1 | uk |
| 16 | 44 | f | 75 | Inflammatory bowel disease | Suicidal | Y | AZA tablets | 2000 | 26.7 | uk |
| 17 | 53 | f | uk | Inflammatory bowel disease, hypertention, peripheral vascular disease | Suicidal | N | AZA tablets | 6000 | 107.5 | uk |
| 18 | 1.5 | m | 11 | None | Domestic | Y | AZA tablets | 25 | 2.3 | 0 |
| 19 | 28 | m | uk | Inflammatory bowel disease, depression | Suicidal | N | AZA tablets | 12500 | 180.1 | uk |
| 20 | adult | m | uk | Unknown diagnosis | Suicidal | N | AZA tablets | 5000 | 72.1 | uk |
| 21 | 39 | m | uk | Inflammatory bowel disease, depression. | Suicidal | N | AZA tablets | 1875 | 27.0 | 75 |
| 22 | 2.5 | m | 13 | None | Domestic | N | AZA tablets | 200 | 15.4 | 0 |
| 23 | 27 | f | 60 | Myasthenia gravis, depression | Suicidal | N | AZA tablets | 1000 | 16.7 | 100 |
| 24 | 15 | f | 44 | None | Suicidal | N | AZA tablets | 250 | 5.7 | 0 |
| 25 | 4 | m | 18 | No underlying condition requiring immunosuppression | Domestic | Y | AZA tablets | 20 | 1.1 | 0 |
| 26 | 2.5 | m | 11 | None | Domestic | Y | AZA tablets | 50 | 4.6 | 0 |
| 27 | 35 | m | 70 | Inflammatory bowel disease | Suicidal | Y | AZA tablets | 15000 | 214.3 | uk |
| 28 | 49 | m | 76 | Inflammatory bowel disease | Suicidal | N | AZA tablets | 2500 | 32.9 | 150 |
| 29 | 79 | f | uk | Unknown condition requiring immunosuppression, end stage renal disease | Iatrogenic | N | AZA tablets | unknown | unknown | uk |
| 30 | 1.4 | m | 11 | None | Domestic | Y | AZA tablets | 150 | 13.6 | 0 |
| 31 | 18 | f | 60 | No underlying condition requiring immunosuppression, previous attempted suicide | Suicidal | N | AZA tablets | 5000 | 83.3 | 0 |
| 32 | 40 | f | uk | Polyarthritis, tramadol dependence | Suicidal | N | AZA tablets | 1000 | 17.9 | uk |
| 33 | 28 | m | 65 | Sensorimotor polyneuropathy | Iatrogenic | Y | AZA tablets | 450 | 6.9 | 150 |
| 34 | 16 | f | 50 | Inflammatory bowel disease | Suicidal | N | AZA tablets | 625 | 12.5 | uk |
| 35 | 21 | m | 60 | Myasthenia gravis | Domestic | Y | AZA tablets | 250 | 4.2 | 125 |
| **Oral Mercaptopurine overdose** | | | | | | | | | | |
| 36 | 3.5 | m | 16 | None | Domestic | Y | 6-MP tablets | 200 | 12.5 | 0 |
| 37 | 3 | f | 20 | Malignancy | Domestic | Y | 6-MP tablets | 150 | 7.5 | 50 |
| 38 | 2 | m | 14 | None | Domestic | Y | 6-MP tablets | 200 | 14.3 | 0 |
| 39 | 1.9 | f | 17 | None | Domestic | Y | 6-MP tablets | 500 | 29.4 | uk |
| 40 | 3.8 | f | 18.4 | Leukaemia | Domestic | Y | 6-MP tablets | 150 | 8.2 | 50 |

^a^ in cases in which patient weight was unknown, the average weight for men or women was used as appropriate (see on-line supplemental methods for further details).

^b^ The details of this case have been published previously [S2]

Abbreviations: m = male, f = female, y = yes, n = no, uk = unknown, AZA = Azathioprine, 6-MP = 6-mercaptopurine
