# Supplementary material for: Acute Thiopurine Overdose: Analysis of Reports to a National Poison Centre 1995–2013
Source: PLoS One. 2014 Jan 29;9(1):e86390. doi: 10.1371/journal.pone.0086390 (PMC3906026; doi:10.1371/journal.pone.0086390)
Supplement: Table S3 — Overdosage expressed as a multiple of the patient's usual dose (or factor above maximum licensed dose), decontamination measures and clinical findings. (DOCX) [file pone.0086390.s004.docx]

**Table S3:** Overdosage expressed as a multiple of the patient’s usual dose (or factor above maximum licensed dose), decontamination measures and clinical findings

| **Patient** | **Multiple of usual or maximum licensed dose** | | **Decontamination measures** | **Clinical findings (within number of days after overdose)** | | | **Severity** | **Relatedness to overdose (likely causeative agent if unrelated)** | |
| --- | --- | --- | --- | --- | --- | --- | --- | --- | --- |
| **Oral Azathioprine overdoses** | | | | | | | | | |
| 1^a^ | 13.00 | | Induction of vomiting by ipecac syrup and single dose charcoal (30g) | | Asymptomatic (14) | |  |  | |
|  |  | |  | | Fall in erythrocyte, leucocyte and thrombocyte counts 2 – 8 days after overdose, but never to below normal | | Minor | Likely | |
| 2 | 10.00 | | None | | Somnolence within 2.25 h | | Minor | Unlikely (midazolam) | |
|  |  | |  | | Confusion within 2.25 h | | Moderate | Unlikely (midazolam) | |
| 3 | 1.50 | | uk; charcoal not recommended | | Unknown | |  |  | |
| 4 | 3.20^b^ | | None | | Asymptomatic (5) | |  |  | |
|  |  | |  | | Blood counts normal (5) | |  |  | |
| 5 | 1.51^b^ | | uk; charcoal recommended | | Altered level of consciousness within 4 h | | Moderate | Unlikely (lorazepam) | |
| 6 | 8.96^b^ | | uk; charcoal not recommended | | Somnolence within 4 h | | Minor | Unlikely (propythendil) | |
|  |  | |  | | Hypotension within 4 h | | Minor | Unlikely (ACE inhibitor and hydrochlorthiazide) | |
| 7 | 0.71^c^ | | Charcoal (0.5 g/kg), repeated after 2h | | Asymptomatic (4) | |  |  | |
|  |  | |  | | Blood count and liver function tests normal (4) | |  |  | |
| 8 | 1.56^b^ | | Uk | | Palpitations within 24 h | | Minor | Possible | |
| 9 | 7.50^b^ | | Charcoal | | Tiredness | | Minor | Unlikely (fexofenadine) | |
| 10 | 7.20^b^ | | Gastric lavage 2 - 3h post overdose | | Asymptomatic (12) | |  |  | |
| 11 | 8.96^b^ | | uk; charcoal recommended | | Nervousness within 2.5 h | | Minor | Unlikely (domperidone, prednisone) | |
| 12 | 8.00 | | uk; charcoal not recommended | | Somnolence within 3 h | | Minor | Unlikely (flupenthixole, melitracen, trimipramine) | |
| 13 | 2.38^b^ | | None | | Somnolence | | Minor | Unlikely (chlorpheniramine, dextramethorphan) | |
|  |  | |  | | Vomiting within 4 h | | Minor | Possible | |
|  |  | |  | | Blood count and liver function tests normal (8) | |  |  | |
| 14 | 9.00 | | None | | Dysarthria | | Moderate | Unlikely (olanzapine) | |
|  |  | |  | | Somnolence | | Minor | Unlikely (olanzapine) | |
|  |  | |  | | Miosis | | Minor | Unlikely (olanzapine) | |
| 15 | 3.22^b^ | | uk, charcoal recommended | | Somnolence | | Minor | Unlikely (haloperidol, diazepam) | |
| 16 | 5.33^b^ | | None | | Sinus tachycardia within 4 h | | Minor | Possible | |
| 17 | 21.50^b^ | | None | | Asymptomatic within 2 h | |  |  | |
| 18 | 0.45^c^ | | Uk | | Asymptomatic within 1 h | |  |  | |
| 19 | 36.02^b^ | | None | | Nausea (2) | | Minor | Certain | |
|  |  | |  | | Abdominal pain (2) | | Minor | Certain | |
|  |  | |  | | Headache (2) | | Minor | Certain | |
|  |  | |  | | Dyspnoea (2) | | Moderate | Certain | |
|  |  | |  | | Increased liver enzymes (10-fold increase in transaminases from baseline) (2) | | Moderate | Certain | |
|  |  | |  | | Fall in leucocyte count (7.2 to 3.9 G/l) (4) | | Minor | Certain | |
| 20 | 14.40^b^ | | Uk | | Agitation | | Minor | Unlikely (alcohol) | |
| 21 | 25.00 | | None | | Dizziness within 2 h | | Minor | Unlikely (mesalazine) | |
|  |  | |  | | Headache within 2 h | | Minor | Possible | |
|  |  | |  | | Vomiting within 2 h | | Minor | Possible | |
| 22 | 3.08^c^ | | Charcoal (1g/kg) | | Asymptomatic within 1 h | |  |  | |
| 23 | 10.00 | | Uk | | Abdominal cramps within 6 h | | Minor | Possible | |
|  |  | |  | | Blood counts and liver function tests normal within 6 h | |  |  | |
| 24 | 1.14^c^ | | uk; charcoal not recommended | | Asymptomatic within 8 h | |  |  | |
| 25 | 0.22^c^ | | uk; charcoal not recommended | | Asymptomatic within 1 h | |  |  | |
| 26 | 0.91^c^ | | uk; charcoal 1g/kg recommended | | Asymptomatic within 1 h | |  |  | |
| 27 | 42.86^b^ | | Single dose charcoal | | Asymptomatic (8) | |  |  | |
|  |  | |  | | Blood counts and liver function tests normal (8) | |  |  | |
| 28 | 16.67 | | None | | Slight reduction in level of consciousness | | Minor | Unlikely (lorazepam) | |
|  |  | |  | | Increased GGT (< 2 x) (8) | | Minor | Likely | |
| 29 | Unknown (Co-therapy with allopurinol) | | uk; Haemodialysis recommended | | Agranulocytosis | | Severe | Certain | |
|  |  | |  | | Sepsis | | Severe | Certain | |
| 30 | 2.73^c^ | | Single dose charcoal 1g/kg | | Asymptomatic (3) | |  |  | |
|  |  | |  | | Blood counts and liver function tests normal (3) | |  |  | |
| 31 | 16.67^c^ | | Single dose charcoal 1g/kg | | Dizziness within 2 h of overdose | | Minor | Unlikely (unknown) | |
|  |  | |  | | Nausea within 2 h of overdose | | Minor | Possible | |
| 32 | 3.58^b^ | | None | | Glasgow Coma Scale score 6 | | Severe | Unlikely (morphine) | |
| 33 | 3.00 | | None | | Asymptomatic (10) | |  |  | |
|  |  | |  | | Blood counts and liver function tests normal (10) | |  |  | |
| 34 | 2.50 (for 2 days) | | None | | Dizziness (2) | | Minor | Unlikely (sertraline) | |
|  |  | |  | | Tremor (2) | | Minor | Unlikely (sertraline) | |
|  |  | |  | | Tiredness (2) | | Minor | Unlikely (sertraline) | |
|  |  | |  | | Elevated bilirubin 42 µmol/l (reference 5 – 26 µmol/l) (2) | | Moderate | Possible | |
|  |  | |  | | Blood counts normal (2) | |  |  | |
| 35 | 2.00 (for 3 days) | | None | | Nausea (3) | | Minor | Certain | |
|  |  | |  | | Abdominal pain (3) | | Minor | Certain | |
| **Oral Mercaptopurine overdose** | | | | | | | | | |
| 36 | 5.00^c^ | Single dose charcoal | | | Asymptomatic (3) |  | | |  |
| 37 | 3.00 | uk, charcoal recommended | | | Asymptomatic within 1 h |  | | |  |
| 38 | 5.71^c^ | None | | | Asymptomatic (1) |  | | |  |
| 39 | 11.76^b^ | Single dose charcoal 1g/kg | | | Asymptomatic (3) |  | | |  |
|  |  |  | | | Blood counts normal (2) |  | | |  |
| 40 | 3.00 | Single dose charcoal 1g/kg | | | Asymptomatic (2 h) |  | | |  |
|  |  |  | | | Liver function tests normal |  | | |  |

^a^ The details of this case have been published previously [S2]. Co-medication at the time of the azathioprine overdose included once weekly subcutaneous methotrexate injections.

^b^ Maximum single licensed dose used to calculate extent of overdose as subject`s usual dose not known

^c^ Maximum single licensed dose used to calculate extent of overdose as subject did not normally receive immunosuppression

G = giga, GGT = gamma glutamyltransferase, h = hour, uk = unknown.
